# Experience of Multitudes

**Published:** 1857-07

**Authors:** 


### EXPERIENCE OF MULTITUDES.

SOME months ago, a young lady from the country, of unusual intelligence and energy, called for medical advice. She had a pulse of a hundred and twenty, a steady bedtime cough, sticking and sore sensation in the throat, tiredness on ascents, unrefreshing sleep, daily pains and soreness about the chest, frequent attacks of sick head ache, fever and night sweats, palpitation of the heart, with other minor symptoms. She attributed her ailment to having gotten her feet wet at a peculiar season, some months before.

During the winter, when the roads in the country were too muddy for walking, and at other times the thermometer was often as low as thirty degrees below zero, she would, rather than forego daily exercise, open both doors of her father's old-fashioned broad hall, and let the wind sweep through it, for some time, until she felt that the air was purified; then closing the doors, she would walk back and forth by the hour, and during the same season, would sleep with a window slightly open, regardless of the weather, and without any fire in the room. On one occasion, when a *city* cousin was on a visit, she allowed herself to have the window down and a fire made, especially as it was a damp, raw, windy, chilly season. The result was, she spent a restless night, and had a copious night sweat.

Seven months later, she writes, not having a single remaining symptom of all those previously named, "The prospect now seems to be, that my health will be permanently restored." The lesson of her letter is, "As I look back a few years, I can now see many things I did which were wrong, and how much I erred in not taking care of my health. Had I done so, it would have saved me much suffering. *Now*, I have learned by experience. Some will learn in that dear school only. I come under that head."

This article presents several strong points, for the mature consideration of all practical and reflecting minds.

